# Ascorbate-Glutathione Cycle and Ultrastructural Analyses of Two Kenaf Cultivars (*Hibiscus cannabinus* L.) under Chromium Stress

**DOI:** 10.3390/ijerph15071467

**Published:** 2018-07-11

**Authors:** Lianmei Niu, Rang Cao, Jingquan Kang, Xu Zhang, Jinyin Lv

**Affiliations:** 1 College of Science, Northwest A&F University, Yangling 712100, China; niulianmei@yeah.net; 2 College of Life Sciences, Northwest A&F University, Yangling 712100, China; xncaorang@nwafu.edu.cn (R.C.); jingquan_kang@163.com (J.K.); zhangxu19930903@163.com (X.Z.)

**Keywords:** chromium, screen, kenaf, AsA-GSH cycle, ultrastructure

## Abstract

Kenaf (*Hibiscus cannabinus* L.) with high tolerance to chromium (Cr) can be used in the phytoremediation of chromium-contaminated soil. However, the mechanisms of chromium accumulation and tolerance in kenaf are still unclear. A hydroponic experiment was taken to screen two kenaf cultivars with Cr tolerance among nine kenaf cultivars via a tolerance index. This is first time the ascorbate-glutathione (AsA-GSH) cycle and chloroplast structural changes involved in Cr tolerance of two kenaf cultivars are explored. This study indicated that enhancement of chromium concentrations reduced nine kenaf growth rates and plant biomass. In addition, in all the nine cultivars, the roots had higher Cr accumulation than the shoots. Cr-tolerant cultivar Zhe70-3 with the maximum tolerant index had the significantly higher enzymatic activities of ascorbate peroxidase (APX), glutathione reductase (GR), dehydroascorbate reductase (DHAR) and mono- dehydroascorbate reductase (MDHAR) in non-enzymatic antioxidant system compared to Cr-sensitive cultivar Zhe77-1. In addition, higher GSH and AsA contents and lower damages of chloroplast ultrastructure were observed in Zhe70-3 under Cr treatment. In conclusion, Cr stress can cause less oxidative stress and destruction of chloroplast ultrastructure in Cr-tolerant cultivar Zhe70-3, and the AsA-GSH cycle may play a crucial role in kenaf Cr tolerance.

## 1. Introduction

Chromium (Cr), an inorganic environmental contaminant in agricultural soil, is non-essential and toxic beyond a certain threshold level [1]. Chromium and its compounds were used in many industries, such as leather tanning, pigment manufacturing, metal finishing, drilling muds and electroplating cleaning agents, all of which can cause environmental Cr contamination [2,3,4]. Hexavalent chromium [Cr (VI)] and trivalent chromium [Cr (III)] are the most stable forms of Cr occurring in soil [5]. Cr (VI) is highly toxic (10–100 times) to plants and humans, compared to Cr (III) [6], but Cr (III) is more stable than Cr (VI) and Cr (VI) is reduced to Cr (III) in soil [1,5]. It has been demonstrated that Cr accumulation in plants can inhibit plant growth leading to decreased root and shoot biomass, affect the key enzymatic activities, induce young leaf chlorosis and change the chloroplast ultrastructure [7,8]. Therefore, due to the seriousness of Cr pollution, it is necessary to identify practicable methods for the remediation of Cr-contaminated soil.

A cost-efficient remediation technology to remove or degrade Cr from contaminated soil, phytoremediation, usually needs high biomass and fast growing plants [9,10]. Kenaf (*Hibiscus cannabinus* L.), with a short and rapid-growing economic fiber herbaceous crop, has been identified for use in phytoremediation [11,12]. As the largest fiber plant worldwide from the Malvaceae family, it is used to the development of adsorbents, high biomass for rope, textiles, fibers in recycled plastics and livestock feed [13,14]. Compared with other hemp species, kenaf has a wide geographical range due to its better ecological adaptability and suitablility for several types of soil [14]. However, little work has been done to improve its phytoremediation abilities by exploring the mechanisms of chromium accumulation and tolerance.

Many studies indicate that one of the major consequences of Cr toxicity is the generation of reactive oxygen species (ROS), which can cause severe damage to plant cells [15,16,17]. Plants can defend themselves against oxidative damage induced by stress through an efficient enzymatic and non-enzymatic antioxidant defense system (the AsA-GSH cycle) to scavenge produced ROS [18,19]. The AsA-GSH pathway regulates ROS levels and modulates redox signaling by changing the activities of key enzymes, including ascorbate peroxidase (APX, EC 1.11.1.11), glutathione reductase (GR, EC 1.6.4.2), monodehydroascorbate reductase (MDHAR, EC 1.6.5.4), ascorbic acid oxidase (AAO, EC 1.10.3.3) and dehydroascorbate reductase (DHAR, EC 1.8.5.1) [20,21]. A previous work revealed that GR and DHAR activities are increased in rice seedlings growing under cadmium stress [22]. AsA and GSH are the major products of the AsA-GSH cycle, and it has been observed that GSH is maintained at a high level under Cr stress [23,24].

Some previous research has reported that kenaf can accumulate heavy metals such as lead (Pb), cadmium (Cd) and copper (Cu) in its shoots and roots, which can affect the growth and antioxidant enzymatic activities [25,26,27]. In addition, we have also reported that Cr stress can alter the activities of some antioxidant enzymes like superoxide dismutase (SOD), catalase (CAT) and peroxidase (POD) in two kenaf cultivars [28]. However, until now there is no report about the effect on the ASA-GSH cycle and ultrastructural changes of kenaf under Cr stress. Therefore, in this study we selected two kenaf cultivars (Cr-tolerant and Cr-sensitive) from among nine cultivars to explore the alterations of key enzymatic activities, non-enzymatic antioxidant contents of AsA-GSH cycle and the ultrastructural changes of the two kenaf cultivars under Cr stress through a hydroponic experiment.

## 2. Materials and Methods

### 2.1. Plant Material and Growth Conditions

Nine cultivars of kenaf seeds, Zhe77-1, Fuhong992, Zhe54-3, Zhe70-3, VG93, Yueyin83-23, Hongyin135, Krasnadoy and T19 were purchased from the Research Institute of Bast Fiber Crops, (Hunan Province, China). Healthy seeds were sterilized with 75% ethanol for 15 min. After repeated rinsing with deionized water, they were soaked for 2 h in sterile water. The seeds were germinated in plastic plots (size: 200 cm × 130 cm × 60 cm, 15 plants per pot) containing sterilized sand in an incubator at 25 °C under a dark condition for three days, then maintained another three days under a 14/10 h light/dark photoperiod.

Afterwards, 15 uniformly germinated seeds were transplanted to similar plastic pots containing 500 mL half-strength modified Hoagland nutrient solution (after adjusting the pH to 6.5). The Hoagland nutrient solution was as described by Hoagland and Arnon [29] and renewed every three days. After seven days, 1.6 mM Cr^3+^ was added into solutions as CrCl_3_·6H_2_O to screen the nine kenaf cultivar seedlings. A Cr-tolerant cultivar (Zhe70-3) and a Cr-sensitive cultivar (Zhe77-1) were selected and exposed to different Cr^3+^ levels (0, 0.5, 1.0 and 1.5 mM). Each chromium treatment was replicated three times. The seedlings were placed in a growth cabinet under 150 μmol m^−2^ s^−1^ photosynthetically active radiation, 14/10 h light/dark photoperiod, 65–75% relative humidity and 25 °C/18 °C day/night temperature. After three days of exposure to Cr^3+^, plants were harvested and used to analyze the effect of chromium on various parameters. Each treatment was replicated three times.

### 2.2. Measurement of Chromium Content

Chromium content was determined as described by Li [30]. A 10 mL acid mixture (HNO_3_:HClO_4_ = 4:1, *v*/*v*) was used to digest the dried samples (about 0.1 g) from the roots and shoots at 220 °C until the liquid became transparent. Subsequently, flame atomic absorbance spectrometry (Hitachi 180-80, Tokyo, Japan) was used to determine Cr content.

### 2.3. Bio-Concentration Factor (BCF), Translocation Factor (TF) and Tolerance Index (TI)

BCF, TF and TI were calculated as described by Shi and Cai [31]. The bio-concentration factor (BCF) is used to describe a plant’s capacity to accumulate Cr, while the translocation factor (TF) indicates the plant’s potential phytoremediation capacity:

BCF = [Cr]_shoot or root_/[Cr]_solution_

TF = [Cr]_shoot_/[Cr]_root_

TI (100%) = [biomass]_Cr_/[biomass]_control_

where [Cr]_solution_ is the concentration of total Cr in solution; [Cr]_shoot_ and [Cr]_root_ are the Cr concentration in the shoot and root parts, respectively. Biomass was expressed using plant growth parameters including the length and mass of roots and shoots.

### 2.4. Measurement of Malondialdehyde (MDA) and Hydrogen Peroxide (H_2_O_2_) Contents

Fresh samples (0.5 g) from the shoots and roots were extracted with 0.5% (*w*/*v*) cold trichloroacetic acid (TCA). After centrifugation (12,000 g, 20 min, 4 °C), the 1/2 supernatant was used to measure the MDA contents, according to the modified method of Li [30]. Another 1/2 supernatant was used to determinate the H_2_O_2_ content as described by Alexieva [32].

### 2.5. Chloroplast Ultrastructural Observation

After three days of Cr treatment, topmost leaf fragments without veins were cut about 0.1 mm × 0.2 mm small species. Then the samples were fixed 12 h in 4% glutaraldehyde (*v/v*) in 0.1 M PBS (phosphate buffer solution, pH 6.8) at 4 °C. After washing in the same PBS, they were post-fixed with 1% OsO_4_ for 2 h at 4 °C and washed in same PBS five times again. Subsequently, with 15-min intervals, an ascending ethanol gradient (30%, 50%, 70%, 80%, 90%, 100%) were used to dehydrate the samples once and 100% ethanol washed twice for 30 min. After embedded in LR-White resin for 12 h, the samples were incubated at 60 °C for two days. Ultrathin sections were obtained with a diamond knife and stained for 8–10 min with 4% Pb. Then morphological parameters were observed via a JEM-1230 transmission electron microscope (JEOL, Tokyo, Japan) at 80 kV.

### 2.6. Measurement of Enzymatic Activities

According to the modified method of Logan and Grace [33], 0.5 g of fresh plant tissues were ground with a chilled mortar and pestle. Then they were extracted with 3.0 mL of 50 mM phosphate buffer (PBS, pH 7.3). This solution containing 0.3% (*w/v*) Triton X-100, 1% (*w/v*) polyvinylpyrrolidone and 1.0 mM Na_2_-EDTA. Enzymes activities were measured after centrifugation (12,000 g, 30 min, 4 °C). The method of Bradford [34] was used to determine protein content, standard curve using bovine serum albumin (BSA) as standard.

APX activity was assayed via measuring the AsA oxidation rate at 290 nm. MDHAR activity was assayed via monitoring the change in absorbance at 340 nm. DHAR activity was assayed via monitoring the absorbance change at 265 nm. A change of 0.01 unit/min was defined as one enzyme unit. The five enzymatic activities were measured following the method of de Pinto [35]. AAO activity was determined by monitoring the change in absorbance at 290 nm according to Esaka [36]. Reduction of 0.01 unit/min was defined as one enzyme unit. GR activity was assayed via monitoring the absorbance change at 340 nm according to Smith [37] and reduction of 0.01 unit/min was defined as one enzyme unit. Each enzyme activity was determined using 1.0 mL total reaction volume.

### 2.7. Measurement of Ascorbate and Glutathione Contents

AsA and total AsA (AsA+DHA) contents were estimated as described following the methods of Zhang and Kirkham [38]. Approximately 0.5 g fresh samples from the shoots and roots were extracted with 3.0 mL pro-cooling 5% (*w/v*) phosphite. After centrifugation (22,000 g, 15 min, 4 °C), the supernatant was used to measure ascorbate content at 525 nm. In order to determine total AsA content, dithiothreitol was added to the supernatant to reduce DHA to AsA. The difference between total AsA and AsA were recorded as DHA content. Total GSH and GSSG contents were estimated as described and modified methods of Zhang and Kirkham [38]. Approximately 0.5 g fresh samples were extracted in 2.5 mL ice-cold 5% sulfosalicylic acid. After centrifugation (KDC-140HR, Zonkia, Hefei, China) (1200 g, 20 min, 4 °C), the half of mixture (supernatant:sulfosalicylic acid = 1:1 *v/v*) was used to determine total GSH (GSH+GSSG) content. Another mixture was pretreated with 2-vinylpyridine to mask GSH before determining GSSG content. The difference between total GSH and GSSG were recorded as GSH content. 

### 2.8. Statistical Analysis

All data were obtained from three replications and analyzed using the SPSS 22.0 software package (SPSS Inc. Chicago, IL, USA) to express the significant differences between mean values at *P* < 0.05 and *P* < 0.01 based on LSD test or Duncan’s multiple range test. All results were presented as means ± standard deviation (SD).

## 3. Results

### 3.1. Screening Cr-Tolerant and Cr-Sensitive Kenaf Cultivars

The toxic effects of chromium on the morphology and productivity are shown in the Supplementary Material in Tables S1 and S2. The chromium treatment significantly decreased the shoot height from 16% to 35.5% as compared with control (Table S1). Moreover, Zhe77-1, in which the decrease of shoot height was 35.5%, was the most sensitive cultivar compared with the others. The root dry mass of all genotypes were insignificantly decreased under Cr stress compared with their controls, respectively, and the reduction rates of Zhe77-1 was 2.5 times that of Zhe70-3 (Table S2). Root Cr concentrations were significantly higher than shoot Cr concentrations in all plant cultivars (Table S3). Meanwhile, the shoot BCF was lower than the root BCF and there was a significant difference between the nine genotypes, except for the T19, Fuhong992 and Hongyin135 cultivars. TF values ranged from 51% to 86% for all cultivars (Table S3), which was used to reflect the capacity of kenaf to translocate Cr from roots to shoots. The subordinate function values of the tolerance index can indicate the degree of plant tolerance, Zhe70-3 had the maximum mean tolerance index of 0.7, while Zhe77-1 had the minimum mean tolerance index of 0.12 (Table 1).

### 3.2. Plant Biomass and Cr Accumulation

As shown in Table 2, under 0.5 mM Cr stress the shoot height and root length of Zhe77-1 significantly decreased by 11.1% and 18.7% compared with their respective controls, while Zhe70-3 had lower values of 8% and 7%. The reduction rates of the root lengths in Zhe77-1 were significantly (11.7%, 17.6% and 24.6%, respectively) higher than these in Zhe70-3 under three different Cr treatments. Similarly, the toxic effects of chromium on dry mass in Zhe70-3 were lower than in Zhe77-1, except under 1.5 mM Cr stress. Although Cr treatment decreased the fresh mass, there were no significant differences between Zhe70-3 and Zhe77-1, except under 1.0 mM Cr stress. Table 2 also shows the Cr accumulation in the two selected plant species under three different Cr treatments, and it was clear that the roots had a higher Cr accumulation than the shoots of both cultivars. With the enhancement of chromium concentrations, Cr contents also increased in the shoots and roots compared with their controls. Under 1.0 and 1.5 mM Cr treatments, the shoot Cr accumulation for Zhe70-3 with 3344 and 4874 mg/kg were higher than 2889 and 4156 mg/kg in Zhe77-1, respectively. All the data show that Zhe77-1 was more Cr-sensitive than Zhe70-3.

### 3.3. Effect of Chromium on MDA and H_2_O_2_ Contents

MDA and H_2_O_2_ contents were increased with the increase in chromium concentrations, and they were higher in the shoots than these in the roots (Table 3). Under 1.5 mM Cr concentration, the contents reached a maximum. The contents of MDA (root) and H_2_O_2_ (shoot/root) appeared to be very similar in Zhe70-3 and Zhe 77-1 at 1.5 mM Cr.

### 3.4. Effect of Chromium on Chloroplast Ultrastructure

**Figure 1:**
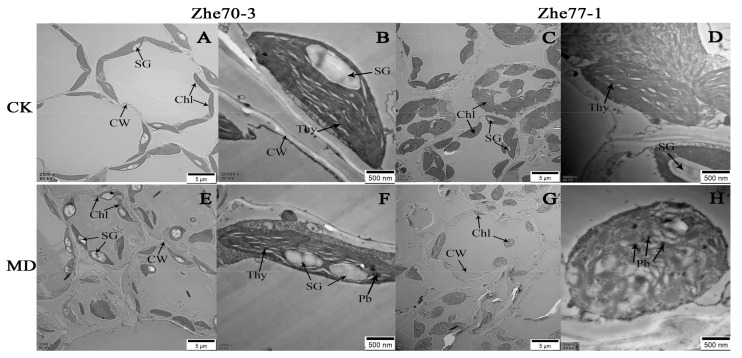
Electron micrographs of leaf mesophyll of Zhe70-3 and Zhe77-1. Plants were exposed to 0 and 1.5 mM Cr stress. (**A**–**D**): TEM micrographs of leaf mesophyll cells of Zhe70-3 and Zhe77-1 under control with low and high magnifications; (**E**–**H**): TEM micrographs of leaf mesophyll cells of Zhe77-1 under 1.5 mM Cr with low and high magnification. Thy: thylakoid, SG: starch grains, Chl: chloroplast, CW: cell wall, Pb: plastoglobuli.

Figure 1 shows the chloroplast ultrastructural changes of kenaf leaf mesophyll under control and 1.5 mM Cr stress in Zhe70-3 and Zhe77-1. The transmission electron microscope (TEM) micrographs reflected that under control condition both cultivars had well-developed chloroplasts (Chl) with thylakoid membranes (Thy) and starch grains (SG) (Figure 1A–D). There were insignificant ultrastructural changes of Zhe70-3 between the control and Cr-treated leaf mesophyll except more SG under 1.5 mM Cr stress, while Zhe77-1 showed disrupted thylakoid membranes and increased plastoglobuli (Pb) compared with their respective controls (Figure 1E,F). In addition, the numbers of chloroplasts were significantly lower compared with control in Zhe77-1. Therefore, it is clear that Zhe70-3 cultivar suffered less damage to the chloroplast ultrastructure compared to the Zhe77-1 cultivar under Cr stress.

### 3.5. Effect of Chromium on Enzyme Activities of AsA-GSH Cycle

**Figure 2:**
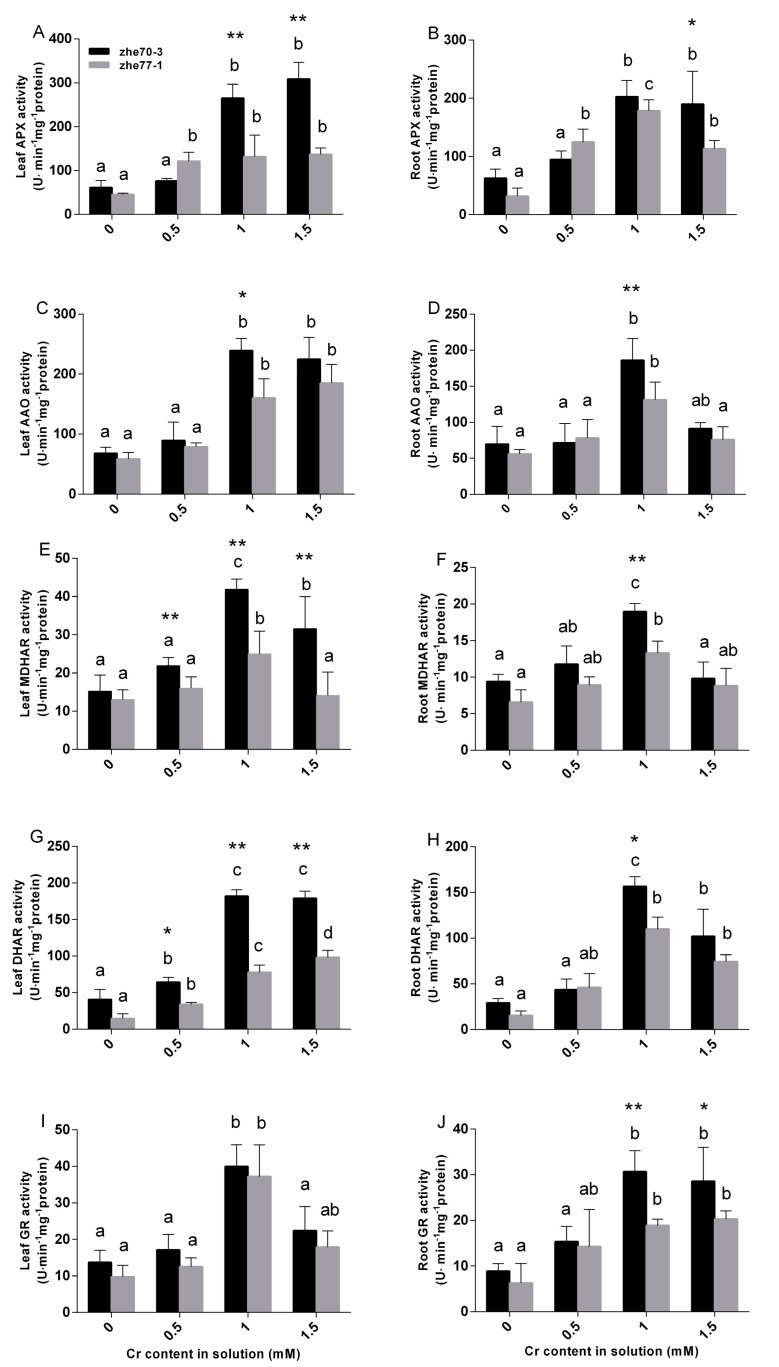
Effects of chromium treatments on enzymatic activities of APX, AAO, DHAR, MDHAR and GR of Zhe70-3 and Zhe77-1. All data show the means ± SD of three replicates. Values followed by the different letters indicate significant differences based on one-way ANOVA followed by LSD test (*P* < 0.05) for each cultivar at different Cr treatments. * and ** indicate separation between the two kenaf cultivars at the same Cr treatment by ANOVA followed by LSD multiple comparison (* *P* < 0.05, ** *P* < 0.01 respectively).

As shown in Figure 2, APX, AAO, GR, MDHAR and DHAR in the AsA-GSH cycle showed different responses to Cr stress and the enzyme activities of the shoots and roots in Zhe70-3 were higher than these in Zhe77-1 (except the shoot GR). 

APX activity in Zhe70-3 shoot was significantly higher than that in Zhe77-1 under 1.0 and 1.5 mM Cr stresses (Figure 2A). However, no significant changes were observed for the root APX activity in the two cultivars (except for 1.5 mM Cr treatment, Figure 2B), while for AAO, there were significant differences in the shoots and roots between the two kenaf cultivars at 1.0 mM Cr stress (Figure 2C,D). MDHAR and DHAR activities presented significant differences at 0.5, 1.0 and 1.5 mM Cr levels in the shoots. However, there were insignificant changes in the roots between both two kenaf cultivars under same Cr stresses except 1.0 mM Cr stress (Figure 2E–H). Increasing Cr concentrations from 0 to 1.5 mM lead to a similar change in GR activity in the shoots and roots compared with controls. The GR activity reached to maximum in Zhe70-3 and Zhe77-1 exposed to 1.0 mM Cr stress, but there were no significant differences in the shoots exposed to same Cr treatments between the two kenaf cultivars (Figure 2I,J). The higher enzyme activities may play an important role for plants to resist the negative effects from Cr. All the results indicated that Cr-tolerant cultivar Zhe70-3 had the higher enzymatic activities, AsA and GSH contents in both shoots and roots than Cr-sensitive cultivar Zhe77-1.

### 3.6. Effect of Chromium on Ascorbate and Glutathione Contents

**Figure 3:**
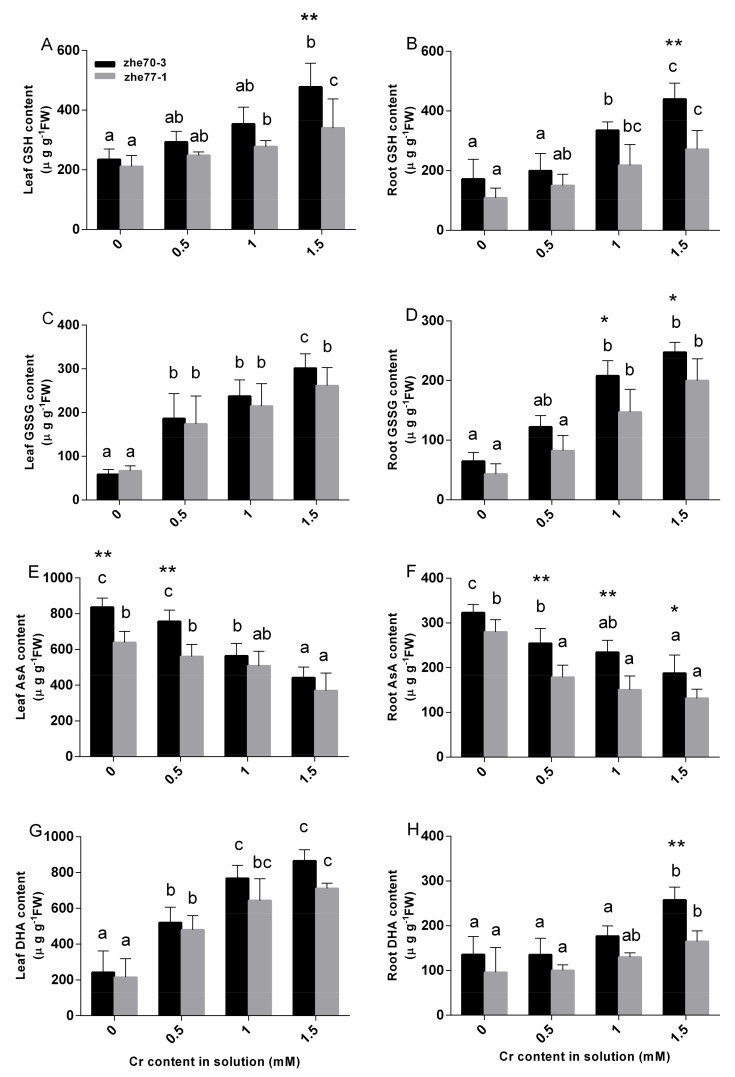
Effect of chromium treatments on GSH and AsA contents of Zhe70-3 and Zhe77-1. All data show the means ± SD of three replicates. Values followed by the different letters indicate significant differences based on one-way ANOVA followed by LSD test (*P* < 0.05) for each cultivar at different Cr treatments. * and ** indicate separation between the two kenaf cultivars at the same Cr treatment by ANOVA followed by LSD multiple comparison (*: *P* < 0.05, **: *P* < 0.01 respectively).

As shown in Figure 3, Cr stress changed the contents of the two major products in AsA-GSH cycle. After exposure to different Cr concentrations, the GSH contents increased, but Zhe70-3 had a significantly higher level both in the shoot and root under 1.5 mM Cr treatment compared to Zhe77-1.

**Figure 4:**
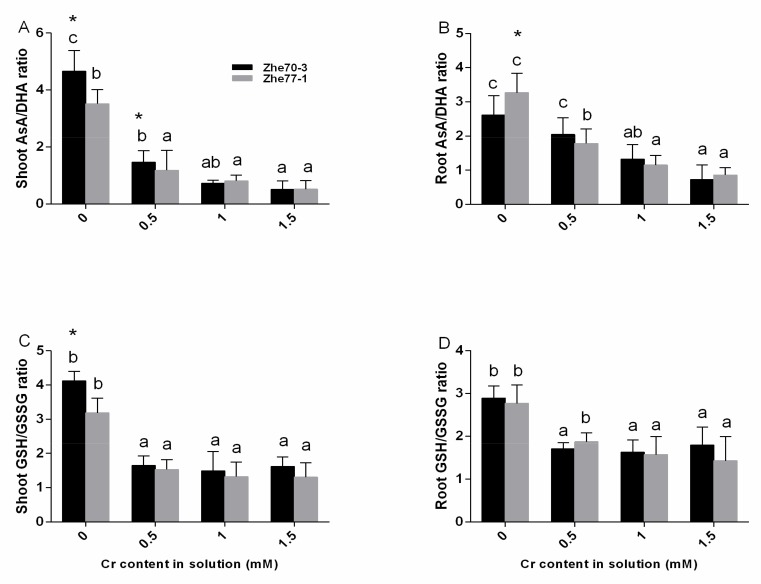
Effect of chromium treatments on AsA/DHA and GSH/GSSG ratio of Zhe70-3 and Zhe77-1. All data show the means ± SD of three replicates. Values followed by the different letters indicate significant differences based on one-way ANOVA followed by LSD test (*P* < 0.05) for each cultivar at different Cr treatments. * indicate separation between the two kenaf cultivars at the same Cr treatment by ANOVA followed by LSD multiple comparison (*: *P* < 0.05).

The GSSG content showed similar increase trends, but there were significant differences in the roots between the two cultivars. In contrast to GSH, AsA contents decreased in both kenaf cultivars with increasing Cr concentrations, and the lowest AsA content was observed at the highest Cr concentration. AsA and DHA contents had different trends in both tissues, roots had a lower level than shoots in both kenaf cultivars and Zhe70-3 were higher compared to Zhe77-1. AsA/DHA and GSH/GSSG ratio showed same decreasing trends in both two cultivars with increasing Cr concentrations, and the minimum values were found at the highest Cr level compared with their respective controls (Figure 4). AsA/DHA values in the shoot were higher than that in the root in two cultivars as well as GSH/GSSG values. All the results suggested that Zhe77-1 was more vulnerable than Zhe70-3 under Cr stress.

## 4. Discussion

Many evidences have demonstrated that Cr is a major ecotoxic heavy metal and high Cr stress inhibits plant growth and productivity [39,40]. Phytoremediation mitigation of metal toxicity has been reported in previous studies [9,10]. Kenaf, is a plant used for phytoremediation, and metal-sensitive and metal-tolerant genotypes have different molecular mechanisms to alleviate heavy metal stress. In this study, a hydroponic experiment was taken to select Cr-sensitive and Cr-tolerant kenaf cultivars according to TI value [31,41]. The Cr-tolerant cultivar Zhe70-3 had the maximum TI value (0.70) and the Cr-sensitive cultivar Zhe77-1 had the minimum TI value (0.12), based on the subordinate function values of TI (Table 1). The reduction in the growth of kenaf by Cr stress might due to Cr-induced generation of ROS as well as chloroplast ultrastructural changes [42,43]. Cr accumulation in roots was at least twice that in shoots in the two kenaf cultivars (Table 2), but compared with Zhe70-3, Cr stress significantly increased the number of starch grains and disrupted the thylakoid membrane in Zhe77-1 (Figure 1). Previously, heavy metal stress on leaf chloroplast ultrastructure was shown in *Moss Taxithelium Nepalense*, *Brassica napus* L. and *Oryza sativa* L. [8,44,45].

To the best of our knowledge, oxidative stress was induced by abiotic stress, resulting in enhanced ROS accumulation. The increased ROS contents may lead to H_2_O_2_ accumulation in chloroplasts and lipid peroxidation indicated by high MDA levels [46,47]. Our data showed that the MDA and H_2_O_2_ contents were lower in Zhe70-3 compared with Zhe77-1, especially Zhe77-1 was much affected after exposure to 1.0 mM Cr concentration, which indicated that it might have undergone stress (Table 3). Plants resist against abiotic and biotic stresses mainly through enzymatic and non-enzymatic antioxidants [18,48]. The AsA-GSH cycle is the most important non-enzymatic system for Cr detoxification [19,49]. In our experiment, Cr stress significantly increased all five measured enzymatic activities (APX, AAO, GR, MDHAR and DHAR) in both tissues of two kenaf cultivars (Figure 2). The results indicated that the Zhe70-3 has higher enzymatic activities (GR, MDHAR and DHAR) in shoots and roots compared with the Zhe77-1 cultivar (Figure 2E–J), which partly showed its tolerance to Cr [27,50,51].

GSH and AsA are the major non-enzymatic antioxidants involved in Cr detoxification [17,50]. A previous study indicated that Cd-tolerant cultivars have higher GSH contents than Cd-sensitive cultivars in rice [52]. Our results showed that the GSH contents in shoots and roots of the Zhe70-3 cultivar was significatly higher than in the Zhe77-1 cultivar under high Cr concentration (Figure 3A,B). Abiotic stress can increase GSH contents by increasing the enzymatic activitives [46,53]. In Figure 3A–D and Figure 4, an increase in GSH and GSSG contents were found in the two kenaf cultivars while the GSH/GSSG ratios declined, similar to previous studies [47,54]. The possible reason may be that DHAR catalyzes the oxidation reaction of GSH to GSSG [55,56]. A negative correlation was observed between the AsA and DHA contents in the two kenaf cultivars. The decreased AsA and increased DHA contents lead to a decline in the AsA/DHA ratio under Cr stress (Figure 3E–H and Figure 4), but higher levels of AsA contents were found in Zhe70-3 compared with Zhe77-1 (Figure 3) as well as the enzymatic activities (Figure 2). These results are in agreement with several previous studies [57,58,59]. All of those may provide the higher Cr-tolerant capacity observed for Zhe70-3.

**Figure 5:**
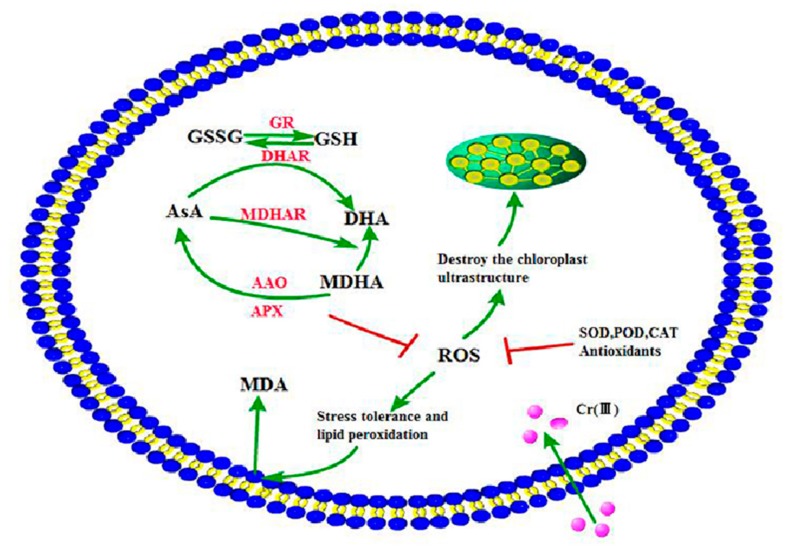
Simplified metabolic and signal transduction pathway of AsA-GSH cycle in regulating Cr accumulation and tolerance.

The AsA-GSH cycle regulates the Cr detoxification of plants via key enzymatic reactions as summarized in Figure 5. The non-enzymatic antioxidant system modulates ROS levels via the enzymes APX, GR, MDHAR, AAO and DHAR. However, more studies are needed to provide more evidence on the molecular mechanisms of AsA-GSH cycle regulating the Cr detoxification in kenaf.

## 5. Conclusions

Generally, our results indicate that Cr was more accumulated in their roots that in shoots in the nine analyzed kenaf cultivars. Chromium treatment decreases the plant biomass, increases the key enzyme activities of MDHAR, DHAR, GR and APX and damages the chloroplast ultrastructure. In addition, compared to the Cr-sensitive cultivar Zhe77-1, the Cr-tolerant cultivar Zhe70-3 has a higher tolerance due to its the higher enzyme activities, ascorbate and glutathione contents of AsA-GSH cycle in response to Cr stress. These results provide an approach to understand the Cr-induced mechanism and Cr-tolerance in kenaf and initiate further studies with dissolved Cr and compare them with the effects in soil systems.

## Figures and Tables

**Table 1:** Subordinate function values of the Tolerance Index of kenaf grown in Cr-treated Hoagland nutrient solution.

Cultivars	Root Length	Shoot Height	Shoot DW	Root DW	Mean	Rank
Zhe70-3	1.154	1.000	0.337	0.310	0.700	1
Yueyin83-23	0.423	0.203	1.000	1.000	0.657	2
Hongyin135	1.000	0.432	0.408	0.103	0.486	3
Zhe54-3	0.808	0.619	0.000	0.379	0.452	4
Fuhong992	0.500	0.751	0.245	0.241	0.434	5
Kransdoy	0.000	0.482	0.735	0.414	0.408	6
VG	0.269	0.482	0.622	0.241	0.404	7
T19	0.539	0.787	0.276	0.000	0.400	8
Zhe77-1	0.115	0.000	0.133	0.241	0.122	9

**Table 2:** Effects of Cr treatments on growth characteristics and Cr content in shoot and root of kenaf cultivars Zhe70-3 and Zhe77-1.

Cultivars	Treatment Cr (mM)	Growth Characteristic				Cr Content (mg kg^−1^ DW)		
		Shoot Height (cm)	Root Length (cm)	Fresh Weight (g)	Dry Weight (g)	Shoot	Root	
Zhe70-3	CK	21.500 ± 1.637e	17.133 ± 1.270d	0.928 ± 0.080e	0.067 ± 0.005c	ND	ND
	0.5	19.733 ± 0.473d	15.933 ± 0.404d	0.710 ± 0.101d	0.061 ± 0.008c	420 ± 0.453a	7897 ± 0.766a
	1	16.633 ± 0.208c	13.167 ± 2.003c	0.563 ± 0.067bc	0.052 ± 0.002b	3344 ± 1.260c	10,467 ± 0.467b
	1.5	15.167 ± 0.321b	12.900 ± 0.700c	0.503 ± 0.078ab	0.045 ± 0.002a	4874 ± 0.897d	11,233 ± 0.645c
Zhe77-1	CK	19.133 ± 0.451d	13.567 ± 0.808c	0.847 ± 0.046e	0.057 ± 0.002c	ND	ND
	0.5	17.000 ± 0.346c	11.033 ± 0.551b	0.648 ± 0.081cd	0.050 ± 0.003b	678 ± 0.178ab	6134 ± 0.269a
	1	16.033 ± 0.416bc	8.467 ± 1.159a	0.486 ± 0.038ab	0.045 ± 0.005a	2889 ± 2.865c	10,345 ± 0.563b
	1.5	12.700 ± 0.656a	7.700 ± 0.458a	0.433 ± 0.033a	0.040 ± 0.002a	4156 ± 0.278d	11,789 ± 1.678c

Biomass and Cr contents of Zhe70-3 and Zhe77-1 under four different Cr stresses. All data show the means ± SD of three replicates. Values with different letters indicate significant differences at the *P* < 0.05 level between treatments. ND: not detect.

**Table 3:** Effects of Cr treatments on MDA and H_2_O_2_ contents in shoot and root of kenaf cultivars Zhe70-3 and Zhe77-1.

Cultivars	Treatment	MDA (mmol·g^−1^ FW)		H_2_O_2_ (µmol·g^−1^ FW)	
	Cr (mM)	Shoot	Root	Shoot	Root
Zhe70-3	CK	0.623 ± 0.271a	0.578 ± 0.042a	0.912 ± 0.213a	0.879 ± 0.092a
	0.5	1.542 ± 0.330ab	0.843 ± 0.359ab	1.348 ± 0.379ab	1.154 ± 0.313ab
	1	2.377 ± 0.219b	1.470 ± 0.650ab	2.791 ± 0.147b	2.532 ± 0.131bc
	1.5	2.775 ± 0.204b	2.656 ± 0.174c	3.917 ± 0.219c	3.214 ± 0.326c
Zhe77-1	CK	0.869 ± 0.102a	0.746 ± 0.233a	1.386 ± 0.132a	0.945 ± 0.147a
	0.5	1.781 ± 0.121ab	1.418 ± 0.347ab	1.731 ± 0.426ab	1.246 ± 0.314ab
	1	2.496 ± 0.253b	2.356 ± 0.195c	2.324 ± 0.202b	1.958 ± 0.420b
	1.5	3.464 ± 0.146c	2.853 ± 0.169c	3.905 ± 0.324c	3.567 ± 0.589c

All data show the means ± SD of three replicates. Values with different letters indicate significant differences at the *P* < 0.05 level between treatments.

## Supplementary Materials

The following are available online at http://www.mdpi.com/1660-4601/15/7/1467/s1, Figure S1: Effects of chromium treatments on growth of two kenaf cultivars (Zhe70-3 and Zhe77-1) in the hydroponic experiment, chromium (Cr^3+^) concentrations were 0, 0.5, 1.0 and 1.5 mM. (a) cultivar Zhe70-3; (b) cultivar Zhe77-1. Table S1: Effects of chromium stress (1.6 mM) on the shoot height and root length of nine kenaf cultivars. Table S2: Effects of chromium stress (1.6 mM) on the dry weight of nine kenaf cultivars. Table S3: Effects of chromium stress on Cr content, BCF and TF in shoot and root of kenaf seedlings. Extremely small amounts of Cr content under control condition in these kenaf materials can be ignored.
