# Supplementary material for: Ascorbate-Glutathione Cycle and Ultrastructural Analyses of Two Kenaf Cultivars (Hibiscus cannabinus L.) under Chromium Stress
Source: Int J Environ Res Public Health. 2018 Jul 11;15(7):1467. doi: 10.3390/ijerph15071467 (PMC6068517; doi:10.3390/ijerph15071467)
Supplement: Supplementary file 1 [file ijerph-15-01467-s001.pdf]

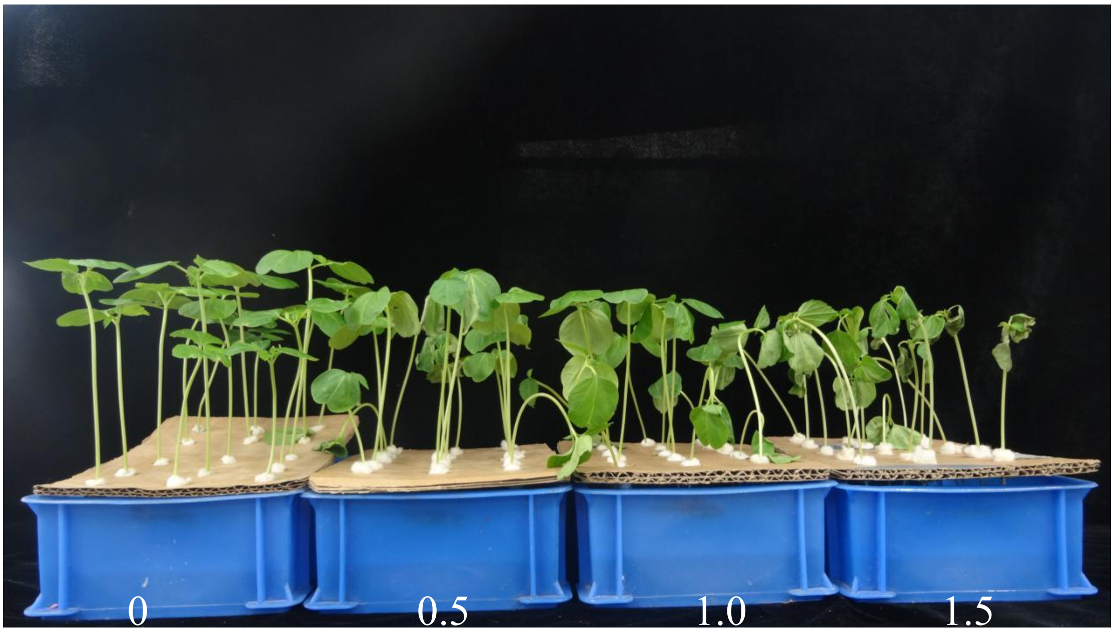

(**a**)

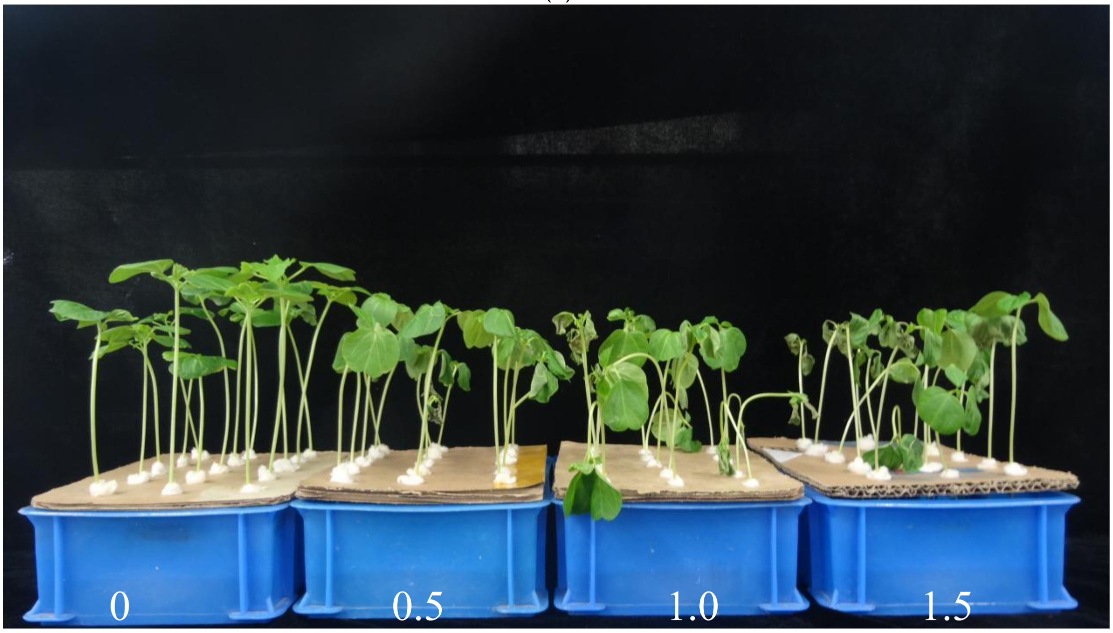

(**b**)

Figure S1. Effects of chromium treatments on growth of two kenaf cultivars (Zhe70-3 and Zhe77-1) in the hydroponic experiment, chromium (Cr^{3+}) concentrations were 0, 0.5, 1.0 and 1.5 mM. (**a**) cultivar Zhe70-3; (**b**) cultivar Zhe77-1.

Table S1. Effects of chromium stress (1.6 mM) on the shoot height and root length of nine kenaf cultivars.

Cultivars	Shoot Height (cm)		Root Length (cm)	
	Control	Cr	Control	Cr
kransdoy	21.12 ± 0.63c	15.10 ± 1.24bcd	12.90 ± 0.79a	11.00 ± 2.42a
VG	19.78 ± 0.58abc	15.10 ± 0.42bcd	12.06 ± 1.01a	11.70 ± 0.84a
Zhe54-3	19.04 ± 0.47ab	15.64 ± 1.20bcd	13.76 ± 1.27a	13.10 ± 0.89a
Zhe77-1	20.48 ± 0.94abc	13.20 ± 0.45ab	12.60 ± 1.52a	11.30 ± 1.44a
Zhe70-3	20.40 ± 0.42abc	17.14 ± 0.97de	15.10 ± 1.78a	14.00 ± 0.61a
T19	20.04 ± 0.79abc	16.30 ± 1.20e	13.90 ± 0.89a	12.40 ± 0.89a
Fuhong992	19.70 ± 1.17ab	16.16 ± 0.23bc	13.10 ± 0.65a	12.30 ± 1.39a
Hongyin135	21.80 ± 0.67c	14.90 ± 1.52bcd	12.26 ± 1.54a	13.60 ± 1.64a
Yueyin83-23	20.76 ± 0.44abc	14.00 ± 0.35bcd	12.08 ± 1.37a	12.10 ± 0.96a

All data show the means ± SD of three replicates. Values with different letters indicate significant differences at the $P < 0.05$ level between treatments, by using the Duncan's multiple range test.

Table S2. Effects of chromium stress (1.6 mM) on the dry weight of nine kenaf cultivars.

Cultivars	Shoot Dry Weight (g/3plants)		Root Dry Weight (g/3plants)	
	Control	Cr	Control	Cr
Kransdoy	0.150 ± 0.004bc	0.103 ± 0.013ab	0.033 ± 0.002de	0.023 ± 0.003abc
VG	0.156 ± 0.008bc	0.112 ± 0.007ab	0.034 ± 0.003ef	0.016 ± 0.002a
Zhe54-3	0.169 ± 0.006c	0.105 ± 0.004ab	0.044 ± 0.001f	0.020 ± 0.002ab
Zhe77-1	0.122 ± 0.005abc	0.116 ± 0.005abc	0.033 ± 0.002de	0.020 ± 0.001ab
Zhe70-3	0.152 ± 0.007bc	0.122 ± 0.007abc	0.026 ± 0.001bcd	0.022 ± 0.002abc
T19	0.107 ± 0.004ab	0.091 ± 0.003a	0.023 ± 0.002abc	0.020 ± 0.001ab
Fuhong992	0.145 ± 0.008abc	0.119 ± 0.004abc	0.030 ± 0.002cde	0.017 ± 0.002ab
Hongyin135	0.154 ± 0.008bc	0.129 ± 0.005abc	0.032 ± 0.003de	0.021 ± 0.002ab
Yueyin83-23	0.161 ± 0.014abc	0.134 ± 0.005abc	0.036 ± 0.003ef	0.021 ± 0.002ab

All data show the means ± SD of three replicates. Values with different letters indicate significant differences at the $P < 0.05$ level between treatments, by using the Duncan's multiple range test.

Table S3. Effects of chromium stress on Cr content, BCF and TF in shoot and root of kenaf seedlings. Extremely small amounts of Cr content under control condition in these kenaf materials can be ignored.

Cultivars	Shoot Cr Content mg·kg^{-1}DW	Root Cr Content mg kg^{-1}DW	Shoot BCF	Root BCF	TF
Kransdoy	5066 ± 208bc	9300 ± 534b	61.06d	111.64e	55%
VG	5033 ± 239bc	8633 ± 289ab	60.72d	103.61c	59%
Zhe54-3	4800 ± 319bc	9333 ± 321b	57.72c	112.43e	51%
Zhe77-1	6066 ± 361c	11366 ± 208c	73.63g	136.95f	53%
Zhe70-3	5566 ± 551c	9200 ± 167ab	67.07f	110.24cd	61%
T19	3666 ± 351ab	7133 ± 493a	44.1a	85.87a	51%
Fuhong992	5400 ± 364bc	9200 ± 819ab	64.96e	110.64de	58%
Hongyin135	4300 ± 361bc	8200 ± 872ab	51.89b	98.57b	53%
Yueyin83-23	2533 ± 187a	8833 ± 205ab	90.96h	106.02c	86%

All data show the means ± SD of three replicates. Values with different letters indicate significant differences at the $P < 0.05$ level between treatments, by using the Duncan's multiple range test.
